# Reviews

**Published:** 1884-05

**Authors:** 


### Reviews.

A YEAR-BOOK OF SURGERY FOR 1883. Edited by CHARLES H. KNIGHT, M.D. 8vo., cloth. pp. 197. New York: G. P. Putnam's Sons, 1884.

This volume gives in clear type a condensed review of the surgical literature of the year, under the following headings: General Surgery and Surgery of the Extremities; of the Head and Neck; of the Chest and Abdomen; of the Genito-Urinary Organs; Venereal Diseases.

Among the interesting matters mentioned are: Sciatica and thorough stretching the nerve by forced flexion of the thigh under complete anæsthesia; permanent wire suture of the olecranon and patella; dislocation of humerus reduced byKocher's method "The elbow joint, flexed at a right angle, is firmly pressed against the side; the arm, still in contact with the body, is slowly and steadily rotated outward until firm resistance is encountered; the arm is then raised forward and a little inward, and lastly, the arm is rotated inward, bringing the hand toward the opposite shoulder;" controlling hæmorrhage in hip-joint operations by rubber bandage; results of several kinds of operations for the excision of the tongue; reports of successful thyrotomy, laryngectomy, pylorectomy, and splenectomy; danger of anæmia after removal of goitre: reduction of obturator hernia; a case of extirpation of rectum, with formation of muscular cutaneous flap preserving the sphincter; supra-pubic cystotomy; spontaneous cure of urethral stricture following obstruction from enlarged prostate.

The book is compiled from several languages, and thus presents a broad view of the surgical arena for the year. Its value need hardly be commented upon.

---

ON THE PATHOLOGY AND TREATMENT OF GONORRHŒA. BY G. L. MILTON. Fifth edition. 8vo., cloth, pp. 306. New York: Wm. Wood & Co., 1884.

This volume of "Wood's Library of Standard Medical Authors" is an abridgment of the writer's earlier editions with the addition of considerable new material, both taken from his contributions to current medical literature, and also prepared expressly for this work.

The author would define gonorrhœa as a specific affection due to an antecedent gonorrhœa, and not the result of infection from other inflammatory processes. "A slight amount of gonorrhœa is more likely to excite the same disease in another person, than a pretty high degree of leucorrhœa is to bring on even simple urethritis."

He is avowedly unscientific, as may be illustrated by the following quotations: In one elderly gentleman, passing a bougie only gently, even though it had been done several times previously without any such result, was followed by slight discharge, etc. "Symptoms apparently as much due to excessive fatigue and thundery weather as to the instrument." We have known an *unclean* instrument to produce these symptoms in perfectly clear weather. Again, gonorrhœal rheumatism, etc., is not considered to be due to systemic infection, but to a "strictly reflex" action. It is not explained what is reflected, or where it is reflected from. Reflex action appears quite often in medical literature, as expressing simply the relation of cause and effect, apparently without consciousness on the part of the writer that it implies a definite process and certainly without the slightest attempt to show how the process is performed.

The author claims that "the tendency of the age is to exalt scientific experiment, however useless it may be, and to pass by the teachings of experience." Surely experiment is simply experience confined in definite channels, and if its many beneficial results have led the profession to distinguish the difference in value between experience and opinion, and if its careful records shall teach the value of the scientific use of language, this " tendency of the age" is hardly to be lamented.

Respecting the study of disease, the author says, and truthfully too, that the interest of medical science required " observation on a simple, uniform plan which dealt only with certainties, which admitted no case as cured or uncured unless the surgeon saw it for himself, and when the history comprehended the beginning and ending of the disease."

The body of the work is made up of a great number of facts and opinions collected from medical literature and from the author's own extensive experience, respecting the various methods of treating the disease and its complications. This is valuable, and worthy of studious attention.

His own theory of treatment is that gonorrhœa can be "cured without the use of the so-called specifics," a proposition that few will deny. Nitrate of silver appears to be his favorite local remedy, both in ordinary cases and in attempted abortive treatment. In one opinion he is firm, that the disease should be vigorously attacked and combatted through its whole course and for some time after the cessation of all symptoms, thus avoiding relapse. The work as a whole is interesting reading, and is the repository of a great amount of valuable information.

---

A YEAR-BOOK OF THERAPEUTICS FOR 1883. Edited by ROYAL W. AMIDON, M.D. 8vo., cloth, pp. 250. $1.50. New York: G. P. Putnam's Sons. 1884. Uniform with Year-Book of Surgery by Knight.

In therapeutics, the year has evidently been one grand raid on the micro-organisms and their germs. How to kill the parasite without killing the host, seems to be the problem put to experimental solution. In tuberculosis of the lung the patients have decidedly got the worst of it. The experiments in Berlin are spoken of as "risky, if not reprehensible." In surgery, corrosive sublimate appears to have come to the front.

The contents of the book are arranged alphabetically, and it presents lengthy papers on Barium Chloride; Corrosive Sublimate; Therapeutic Means Directly Affecting the Local Processes of Inflammation; Physiological Action of Iodoform; Death from Methylene, and Psoriasis.

Among the minor items may be mentioned the compressed air douche; the use of amyl nitrite in congestive chill; arsenic a specific for idiopathic anæmia; production of ozone in the air by the diffusion of oil of turpentine; aseptic, straight glass catheter, with a bevelled opening, as prophylaxis of cystitis in women; kairine to reduce temperature—lastly, the parasitic origin of pertussis, resorcin being the proper germicide. C. C. W.

---

ECZEMA AND ITS MANAGEMENT. By L. DUNCAN BULKLEY, A.M., M.D. Second Edition, 8vo., cloth, pp. 344. New York: G. P. Putnam's Sons. 1884.

This handsomely prepared volume contains much practical instruction relative to the management of the disease. In the last chapter there are seventy-three prescriptions for use in its treatment. As will be seen by his definition, the author lays great stress on itching as a characteristic of the disease, and excludes all inflammations of the skin that are the result of external irritation. "A non-contagious, inflammatory disease of the skin, of constitutional origin, acute or chronic in character, manifesting any or all the results of inflammation at once or in succession, and accompanied by burning and itching." In respect to the constitutional origin, the author considers that all cases arise either from a scrofulous, gouty, or neurotic state. He wastes much ink and paper in explaining these diatheses, and the practical outcome of the whole matter is that the constitutional cause of eczema is undiscovered.

A diathesis is something from its very nature intangible and indescribable; it never can be diagnosticated during health, for healthy people do not apply to a physician, and after the invasion of any disease the general features of the case show no longer a diathesis, a predisposition to the disease, but a cachexia, the results of that disease. If the diathesis could be diagnosticated during health, it would do no good, for there are many diseases ascribed to each diathesis, and the patient, his condition being inherited and not amenable to treatment, would live in constant fear, not knowing at what point to guard himself. If this ghost of demonology could be exorcised, and men would be content to study diseases and their treatment, rather than the figments of their own imaginations, humanity would be the gainer by it.

---

THE INTERNATIONAL ENCYCLOPÆDIA OF SURGERY. Edited by JOHN ASHHURST, JR., M.D. Illustrated with chromo-lithographs and wood-cuts. In six volumes. Vol. IV, 8vo, cloth, 987 pp. New York: William Wood & Co. 1884.

This volume contains the following articles:

Injuries of Bones, by John H. Packard, M.D., a systematic and exhaustive study of the fractures of the bones and cartilages and their treatment. It occupies 260 pages, and contains 49 wood-cuts.

Diseases of the Joints, by Richard Barwell, F.R.C.S., 177 pages and 46 wood-cuts. This paper is written in a pleasing style, which gratifies the ear, although it may obscure the sense.

The author's idea of pathology may be fairly illustrated by the following extract: There are two varieties of struma. In one "the head is large and uneven, big behind; the ears are large and stand out widely; the undecided, thick, under lip is apt to droop; * * * add to this a dull, coarse hair, gravel-colored or lusterless black," and we have the type which tends to the scrofulous diseases. "The characteristics of the other kind are much more pleasing, indeed often exquisitively beautiful; the thin skin, almost translucent in its delicacy, either of the milk-and-rose or of the soft brunette complexion, covers features of the finest form and most refined modelling; the long eye-lashes fringe their lids marked with a delicate trac ery of wandering veins;" etc., etc. This type tends to tuberculosis. All this is very nice on paper, but it leaves so broad a ground for differences of opinion that it becomes simply a matter of opinion, and is hardly worthy of a place among scientific facts.

It would appear from our author's description, that only those children were strumous who are particularly ugly or particularly beautiful.

Excisions and Resections, by John Ashurst, Jr., M.D., 96 pages and 77 wood-cuts. This paper contains the descriptions of the operations and the tabulated results of many hundred cases.

Excision of the Knee-Joint, by George E. Fenwick, M.D., C.M., a short paper giving a table of twenty-eight cases.

Tumors, by Henry Trentham Butlin, F.R.C.S., 113 pages, several wood-cuts and four good lithographic plates, showing 31 figures of microscopic preparations. There are also several horrible colored representations of tumor, *in situ*. They are neither artistic in finish or exact in detail. The text is clear in its descriptions and conveniently arranged for reference.

Injuries of the Back, by John A. Lidell, A.M., M.D., 223 pages, four wood-cuts. Nearly one-half of this lengthy paper consists of the reports of cases largely from military practice. In many instances these quotations are from careless observers, and often they poorly illustrate the topics under which they are cited. Speaking of the temporal and carotid arteries, he thus quotes from one author: "In fact, the action of these vessels coincided in neither force or *frequency* with that of the radial and ulnar arteries." The only explanations that occur to us for this, is that either there was marked dicrotism of the pulse in one set of vessels and not in the other, or, what is more probable, that the observer was grossly at fault. We would respectfully engage that the New York *Sun*, vid. page 716, while it may be never so reliable an authority as to matters of fact, should hardly be quoted in matters of opinion requiring special surgical knowledge. The writer strongly advocates the immediate reduction of dislocations, with or without fracture of the spine. He fully illustrates the fallacy of expectant treatment, as well as the strikingly beneficial results of active surgical interference. Respecting

*concussions* of the spinal cord he is again very unfortunate in his quotations.

In the most striking illustration given is the following expression "Autopsy. * * * The substance of the cord was *contused* opposite the fourth and fifth cervical vertebræ." This is on page 790, while on page 794 the same case is quoted from Bryant, and credited to Guy's Hospital Reports, 3rd series, vol. V, it should have been vol. IV, as illustrating *contusion* of the cord. This confusion is evidently the result of careless compilation. The author points out the danger of inflammation of the cord and its membranes, arising from traumatic causes, and gives lengthy descriptions of the secondary results of injury to this organ, as sacro-gluteal eschars, disorders of the urinary organs, etc.

Malformations and Diseases of the Spine, Frederic Treves, F.R.C.S. This concluding paper occupies 72 pages, and has a few cuts. It is far from exhaustive, and is particularly defective in its consideration of the treatment of Pott's Disease, the theory and application of the antero-posterior splint being briefly and poorly stated, while the plaster of paris splint, an appliance having a much narrower range of usefulness, is unnecessarily enlarged upon. C. E. W.

---

A WOMAN WITH FOUR MAMMARY GLANDS.—Dr. W. E. Whitford, of Rossie, N. Y., writes that he was recently called to see a woman, aged 38, mother of five children, who was suffering from an abscess in one of her breasts. About three inches below the nipple on each side, there were miniature mammary glands. After confinement these would become quite large, and secrete milk for about two months.

